# Pirfenidone attenuates synovial fibrosis and postpones the progression of osteoarthritis by anti-fibrotic and anti-inflammatory properties in vivo and in vitro

**DOI:** 10.1186/s12967-021-02823-4

**Published:** 2021-04-19

**Authors:** Qilu Wei, Ning Kong, Xiaohui Liu, Run Tian, Ming Jiao, Yiyang Li, Huanshuai Guan, Kunzheng Wang, Pei Yang

**Affiliations:** Bone and Joint Surgery Center, The Second Affiliated Hospital of Xi’an Jiaotong University, Xi’an, 710004 China

**Keywords:** Osteoarthritis, Pirfenidone, Human fibroblast-like synoviocytes, Synovium, Fibrosis, Inflammation

## Abstract

**Background:**

Osteoarthritis (OA) is a disease of the entire joint involving synovial fibrosis and inflammation. Pathological changes to the synovium can accelerate the progression of OA. Pirfenidone (PFD) is a potent anti-fibrotic drug with additional anti-inflammatory properties. However, the influence of PFD on OA is unknown.

**Methods:**

Proliferation of human fibroblast-like synoviocytes (FLSs) after treatment with TGF-β1 or PFD was evaluated using a Cell Counting Kit-8 assay and their migration using a Transwell assay. The expression of fibrosis-related genes (*COL1A1*, *TIMP-1*, and *ACTA-2*) and those related to inflammation (*IL-6* and *TNF-α*) was quantified by real-time quantitative PCR. The protein expression levels of COL1A1, α-SMA (coded by *ACTA-2*), IL-6 and TNF-α were measured by enzyme-linked immunosorbent assay. A rabbit model of OA was established and then PFD was administered by gavage. The expression of genes related to fibrosis (*COL1A1*, *TIMP-1*, and *ADAM-12*) and inflammation (*IL-6* and *TNF-α*) was measured using RNA extracted from the synovium. Synovial tissue was examined histologically after staining with H&E, Masson’s trichrome, and immunofluorescence. Synovitis scores, the volume fraction of collagen, and mean fluorescence intensity were calculated. Degeneration of articular cartilage was analyzed using a Safranin O-fast green stain and OARSI grading.

**Results:**

The proliferation of FLSs was greatest when induced with 2.5 ng/ml TGF-β1 although it did not promote their migration. Therefore, 2.5 ng/ml TGF-β1 was used to stimulate the FLSs and evaluate the effects of PFD, which inhibited the migration of FLSs at concentrations as low as 1.0 mg/ml. PFD decreased the expression of COL1A1 while TGF-β1 increased both mRNA and protein expression levels of IL-6 but had no effect on α-SMA or TNF-α expression. PFD decreased mRNA expression levels of *COL1A1*, *IL-6*, and *TNF-α *in vivo. H&E staining and synovitis scores indicated that PFD reduced synovial inflammation, while Masson’s trichrome and immunofluorescence staining suggested that PFD decreased synovial fibrosis. Safranin O-Fast Green staining and the OARSI scores demonstrated that PFD delayed the progression of OA.

**Conclusions:**

PFD attenuated synovial fibrosis and inflammation, and postponed the progression of osteoarthritis in a modified Hulth model of OA in rabbits, which was related to its anti-fibrotic and anti-inflammatory properties.

## Background

Osteoarthritis (OA) is the most common joint disease in the elderly and represents one of the most important causes of disability [1]. Current research suggests that OA is a disease of the whole joint involving the cartilage, subchondral bone, ligaments, meniscus, in addition to the synovium [2]. The synovium is a thin membranous tissue enclosing diarthrodial joints, and secretes compounds such as hyaluronate and lubricin which facilitate normal joint function [2, 3]. Synovial inflammation and fibrosis triggered by OA can lead to chondrocyte apoptosis, and accelerate cartilage damage, exacerbating joint dysfunction that ultimately leads to pain and joint stiffness [4, 5]. Therefore, elucidation of the role of synovial pathological changes that occur as OA progresses may provide new strategies for future therapies of OA.

Synovial inflammation is characterized by inflammatory cell infiltration and the production of proinflammatory cytokines such as tumor necrosis factor-α (TNF-α), interleukin-6 (IL-6), and interleukin-1 (IL-1) [6]. Synovial fibrosis is defined as the excessive secretion of extracellular matrix components including collagen type I (coded by human collagen type 1 α 1, *COL1A1*), fibronectin, or thrombospondin [7]. Transforming growth factor-β (TGF-β) signaling plays a key role in fibrosis [7, 8]. Briefly, TGF-β initially binds to its type II receptor and then recruits two type I receptors, activin receptor-like kinase 5 (ALK-5) and ALK-1. The complex subsequently phosphorylates the cytoplasmatic receptor-regulated SMADs (R-SMADs), SMAD-2/3 and SMAD-1/5/8. R-SMADs bind SMAD-4 (also termed common SMAD) prior to translocation to the nucleus where regulation of target gene transcription occurs [7]. In addition to TGF-β, other factors, including tissue inhibitor of metalloproteinase 1 (TIMP-1), a disintegrin and metalloproteinase domain-containing protein 12 (ADAM-12) and alpha-smooth muscle actin (α-SMA, encoded by the pro-fibrotic marker alpha smooth muscle actin 2, *ACTA-2*) are also reported to be elevated in synovial fibrosis [2, 9].

Pirfenidone (5-methyl-1-phenyl-2-(1H)-pyridine, PFD) is an anti-inflammatory and anti-fibrotic drug. It is widely used in clinical practice for idiopathic pulmonary fibrosis [10, 11]. Its anti-fibrotic properties have been evaluated in animal models of lung injury [12, 13], kidney injury [14], and liver fibrosis [15], etc*.* Previous studies have established that PFD significantly suppresses TGF-β1-induced fibrosis [16, 17] and decreases the expression of inflammatory factors including TNF-α and IL-6 [18]. However, whether PFD can influence synovial inflammation or fibrosis in OA still requires additional investigation.

In the present study, TGF-β1-stimulated fibroblast-like synoviocytes (FLSs) isolated from patients with osteoarthritis were evaluated, to elucidate the anti-fibrotic and anti-inflammatory properties of PFD. In addition, an OA model in domestic rabbits was established to investigate whether pirfenidone attenuates fibrosis of the synovium and postpones the progression of osteoarthritis. We anticipate the findings of the study will provide important insights that facilitate the development of synovium-targeting therapy in OA.

## Methods

### Isolation of FLSs

Samples of synovial tissue were isolated from 3 patients with OA (mean age: 60 ± 4 years; range: 56–64 years) who were undergoing total knee arthroplasty (TKA). The patients fulfilled the revised criteria of the American College of Rheumatology for OA [19]. Informed consents were obtained from all patients. The experimental protocol was approved by the Human Research Ethics Committee of the Second Affiliated Hospital of Xi’an Jiaotong University.

FLSs were isolated by enzymatic digestion of the synovial tissue obtained from patients with OA undergoing total knee arthroplasty (TKA), as described previously [20]. Briefly, specimens were minced into small pieces and then digested with 4 mg/ml type I collagenase (Solarbio, Beijing, China) for an hour at 37 °C within an atmosphere containing 5% CO_2_. After digestion, the isolated FLSs were filtered using a 70 μm cell filter after which the cells were centrifuged and washed three times with phosphate buffered saline (PBS). The FLSs were cultured in Dulbecco’s modified Eagle’s medium (DMEM, Gibco, NY, USA) supplemented with 10% fetal bovine serum (FBS, Gibco, NY, USA), 100 IU/ml penicillin, and 100 IU/ml streptomycin (Gibco, NY, USA). The cells were maintained at 37 °C in a humidified atmosphere containing 5% CO_2_. The medium was replaced every 3 days with fresh culture medium. The cells were digested with 0.25% trypsin and passaged when 80% confluent, at a ratio of 1:3 in fresh culture flasks. Passage 3 to 8 cells were used for experimentation.

### Cell proliferation assay

Cell proliferation was determined using a Cell Counting Kit-8 assay (CCK-8; Dojindo Laboratories, Kumamoto, Japan) in accordance with the manufacturer’s instructions. Recombinant human TGF-β1 was obtained from Abcam (Cambridge, UK) and dissolved in PBS prior to use. FLSs were allocated into PFD or TGF-β1 groups. Cells from each group were plated as replicates of three in 96-well plates (5 × 10^3^ cells/well) in 100 μL DMEM supplemented with 10% FBS and incubated overnight to allow them to adhere. Subsequently, cells in the PFD group were treated with 0 (control group), 0.5, 1.0, 1.5, or 2 mg/ml PFD for 24, 48, and 72 h. The TGF-β1 group was treated with 0 (control group), 1, 2.5, 5, 8, or 10 ng/ml TGF-β1 for 24, 48, and 72 h. Ten μL CCK-8 solution were then added to each well and incubated for 2 h at 37 °C. The optical density (OD) at 450 nm was measured using a microplate reader (S/N 415–2687, Omega Bio-Tek, Ortenberg, Germany). Inhibition of cell growth rate was calculated using the following formula: [(OD value of control–OD value of PFD-treated cells)/OD value of control] × 100%. The increase in cell growth rate was calculated as follows: [(OD value of TGFβ1-treated cells–OD value of control)/OD value of control ]×100%.

### Migration assay

From the results of the cell proliferation assay, 2.5 ng/ml TGF-β1 stimulated the greatest proliferation rate in FLSs and so this concentration was used for subsequent experiments.

To evaluate migration, FLSs (10^5^/well) were cultured in the upper chamber of Transwell inserts with an 8 μm pore size (Transwell, Corning, NY, USA) and then treated with PFD at concentrations of 0 (control group), 0.5, 1.0, 1.5, or 2 mg/ml, or with 2.5 ng/ml TGF-β1 in serum-free DMEM for 24 h. Cells on the upper surface of the filters were removed and those on the underside counted using ImageJ software after staining with crystal violet. Each experiment was repeated 3 times.

### ELISA

To eliminate the influence of PFD on proliferation, FLSs (10^6^/well) were initially cultured in 6-well plates in 2.5 ml DMEM supplemented with 10% FBS for 72 h or until they reached 80% confluency. The FLSs were then treated with PFD (0, 0.5, 1.0, 1.5, or 2 mg/ml), or with 2.5 ng/ml TGF-β1 for 72 h. The conditioned medium was collected and levels of TNF-α, IL-6, and COL1A1 quantified using the respective enzyme-linked immunosorbent assay (ELISA) kits (Jianglai, Shanghai, China). The absorbance at 450 nm was measured using a microplate reader (S/N 415–2687, Omega Bio-Tek, Ortenberg, Germany).

### Surgical osteoarthritic model and groups

All animal experiments were approved by the institutional animal care and use committee of Xi’an Jiaotong University.

A total of 20 six-month-old domestic rabbits weighing approximately 2.5–3 kg were bought from the animal center of the Medical School of Xi’an Jiaotong University. They were allocated randomly into 4 groups: sham control group (Ctrl), 4-week group, 12-week group, and PFD group (PFD) (Table 1). The rabbit model of OA was induced in all groups except for the control, using a modified version of the Hulth method. Briefly, the rabbits were anesthetized by injection of 30 mg/kg sodium pentobarbital (Sigma-Aldrich, Saint Louis, USA) into the ear vein and then placed in a supine position. The skin over the right knee joint was shaved and then disinfected. After administration of the anesthesia, a 4 cm anteromedial incision was created and the medial collateral ligament transected. The articular cavity of the right knee was then exposed and the medial meniscus excised. The anterior cruciate ligament was then transected. Medial–lateral stress and anterior drawer tests were performed. Positive tests indicated that the surgery was successful. Hemostasis was achieved after which the joint was washed with sterile saline. The surgical incision was then closed layer-by-layer after which the rabbits were placed in individual cages to recuperate. A total of 400,000 U sodium penicillin (North China Pharmaceutical Co Ltd, Hebei, China) was administered intramuscularly for 3 consecutive days postoperatively.

**Table 1:** Information of the 4 groups

Groups	Number	Hulth’s method	Pirfenidone
Sham control group (Ctrl)	5	No	No
4-week group	5	Yes	No
12-week group	5	Yes	No
PFD group (PFD)	5	Yes	Yes

For the sham control group (Ctrl), a 4 cm anteromedial incision only was created, after which it was closed by suturing. Validity of the modified Hulth model was assessed in the 4 and 12-week groups. At each time point, 5 rabbits were sacrificed using an overdose of anesthesia. Each rabbit in the PFD group received PFD at 30 mg/kg body weight every day for 4 weeks by gavage since the 4th week after surgery. This dosage was established in a previous study on rabbits [21]. The rabbits of the PFD group and sham control groups were sacrificed at the 12th weeks using an overdose of anesthesia. The cartilage from the medial femoral condyle and the synovium located between the patella and femur were collected for subsequent analysis.

### Real-time quantitative PCR

Total RNA was extracted from rabbit synovial tissue using TRIzol reagent (Takara, Dalian, China) and from FLSs treated with the different interventions (cultured in DMEM with 0, 0.5, 1.0, 1.5, or 2 mg/ml PFD with or without 2.5 ng/ml TGF-β1 for 72 h) in accordance with the manufacturer’s instructions. A 1 µg quantity of total RNA was reverse transcribed into cDNA using oligo(dT) primers using an M-MLV First Strand cDNA Synthesis kit (Omega, Guangzhou, China) as follows: 18 μl reaction mixture containing 1 μg of total RNA, 1 μl oligo d(t), 1 μl dNTP mix and nuclease-free water. The reaction mixture was incubated at 70 °C and then immediately transferred to an icebox. A 5 μl aliquot of 5 × RT buffer, 1 μl M-MLV reverse transcriptase, and 1 μl RNase inhibitor were added and the mixture was then allowed to react at 42 °C for 60 min and then finally terminated at 85 °C for 5 min. Real-time quantitative PCR (RT-qPCR) analysis was performed using 2 × Universal SYBR Green Fast qPCR mix (ABclonal, Wuhan, China) as follows: 2 min at 95 °C, then 40 cycles of 5 s at 95 °C and 30 s at 60 °C. RT-qPCR assays were conducted in triplicate in a Bio-Rad system (CFX Connect, California, USA). Cycle threshold (Ct) values were recorded for each gene and target mRNA levels normalized to the glyceraldehyde 3-phosphate dehydrogenase (GAPDH) level. Relative gene expression was calculated using the 2^−ΔΔCt^ method. The relevant primer sequences are shown in Table 2.

**Table 2:** Experimental correlation primers

Gene	Primer sequence
Human-GAPDH	Forward-5′-GGGCTCTCCAGAACATCATCC-3′
	Reverse-5′-GTCCACCACTGACACGTTGG-3′
Human-COL1A1	Forward-5′-ACGAAGACATCCCACCAATC-3′
	Reverse-5′- AGATCACGTCATCGCACAAC-3′
Human-TIMP-1	Forward-5′-CTTCTGCAATTCCGACCTCGT-3′
	Reverse-5′-ACGCTGGTATAAGGTGGTCTG-3′
Human-ACTA-2	Forward-5′-GCGTGGCTATTCCTTCGTTA-3′
	Reverse-5′-GGCAACTCGTAACTCTTCTCAA-3′
Human-TNF-α	Forward-5′- TAAGCAACAAGACCACCACTTC-3′
	Reverse-5′- TCTCCAGATTCCAGATGTCAGG
Human-IL-6	Forward-5′-ACTCACCTCTTCAGAACGAATTG-3′
	Reverse-5′-CCATCTTTGGAAGGTTCAGGTTG-3′
Rabbit-GAPDH	Forward-5′-GAAGGTGGTGAAGCAGGCATCC-3′
	Reverse-5′-GGCACTGTTGAAGTCGCAGGAG-3′
Rabbit-COL1A1	Forward-5′-TGGCGAGCCTGGAGCTTCTG-3′
	Reverse-5′-GCTTCTCCGTCATCTCCGTTCTTG-3′
Rabbit-TIMP-1	Forward-5′-AGGCTCTGACAAGGGCTTCCAG-3′
	Reverse-5′-GGTGTAGGCTTCGGCTTCCAAC-3′
Rabbit-ADAM-12	Forward-5′-CCGAACCTTGACCTTGAG-3′
	Reverse-5′-ACCACCACCTTCCTATTCT-3′
Rabbit-IL-6	Forward-5′-GGCTGATAGAAGAAGACGGATG-3′
	Reverse-5′-CCATGCCTGTCCAGAGATAAAG-3′
Rabbit-TNF-α	Forward-5′-CCTTCCTCTCCTCAGATGTTTC-3′
	Reverse-5′-ACGGGTCAGTCACCAAATC-3′

### Histological analysis

Cartilage from the medial femoral condyle and the synovium harvested from between the patella and femur were fixed in 4% paraformaldehyde overnight. Cartilage tissues were decalcified in 13% ethylenediaminetetraacetic acid (EDTA) for 4 weeks. Those tissues were then dehydrated through an increasing gradient of ethanol concentrations and permeabilized in xylene, then finally embedded in paraffin wax. The paraffin blocks were sliced into 5-μm sections in the sagittal plane using a microtome. Cartilage was analyzed using Safranin O-Fast Green staining, while H&E, immunofluorescence, and Masson’s trichrome staining were performed on the synovium.

Degeneration of the articular cartilage was assessed after Safranin O-Fast Green staining using the Osteoarthritis Research Society International’s histopathology grading system (OARSI). Cartilage degeneration was scored as absent to severe (in numerical values 0–24), as described previously [22]. Synovitis was identified through H&E staining and scored in accordance with principles published in a previous study [23].

Masson’s trichrome and immunofluorescence staining were used to estimate the severity of synovial fibrosis. For immunofluorescence staining, anti-COL1A1 antibody (NB600-450, Novus, Colorado, USA) was used as the primary antibody and Cyanine 3 conjugated goat anti-mouse IgG (GB21301, Servicebio, Wuhan, China) as the secondary antibody. Nuclei were counterstained using DAPI (4′,6-diamidino-2-phenylindole-dihydrochloride, G1012, Servicebio, Wuhan, China). Staining was analyzed using a laser scanning confocal microscope (Nikon Eclipse C1, Tokyo, Japan). The volume fraction of collagen in Masson’s trichrome stained sections and mean fluorescence intensity of immunofluorescence stained sections were calculated using ImageJ software and used to analyze the severity of synovial fibrosis. All sections were analyzed by 2 observers blinded to the study.

### Statistical analysis

Data are presented as means ± SD of three independent experiments. Statistical differences between treatment groups were calculated using an unpaired Student’s t-test using SPSS v24.0 software. P-values < 0.05 were considered statistically significant.

## Results

### Effects of PFD or TGFβ1 treatment on the proliferation and migration of FLSs

**Fig. 1:**
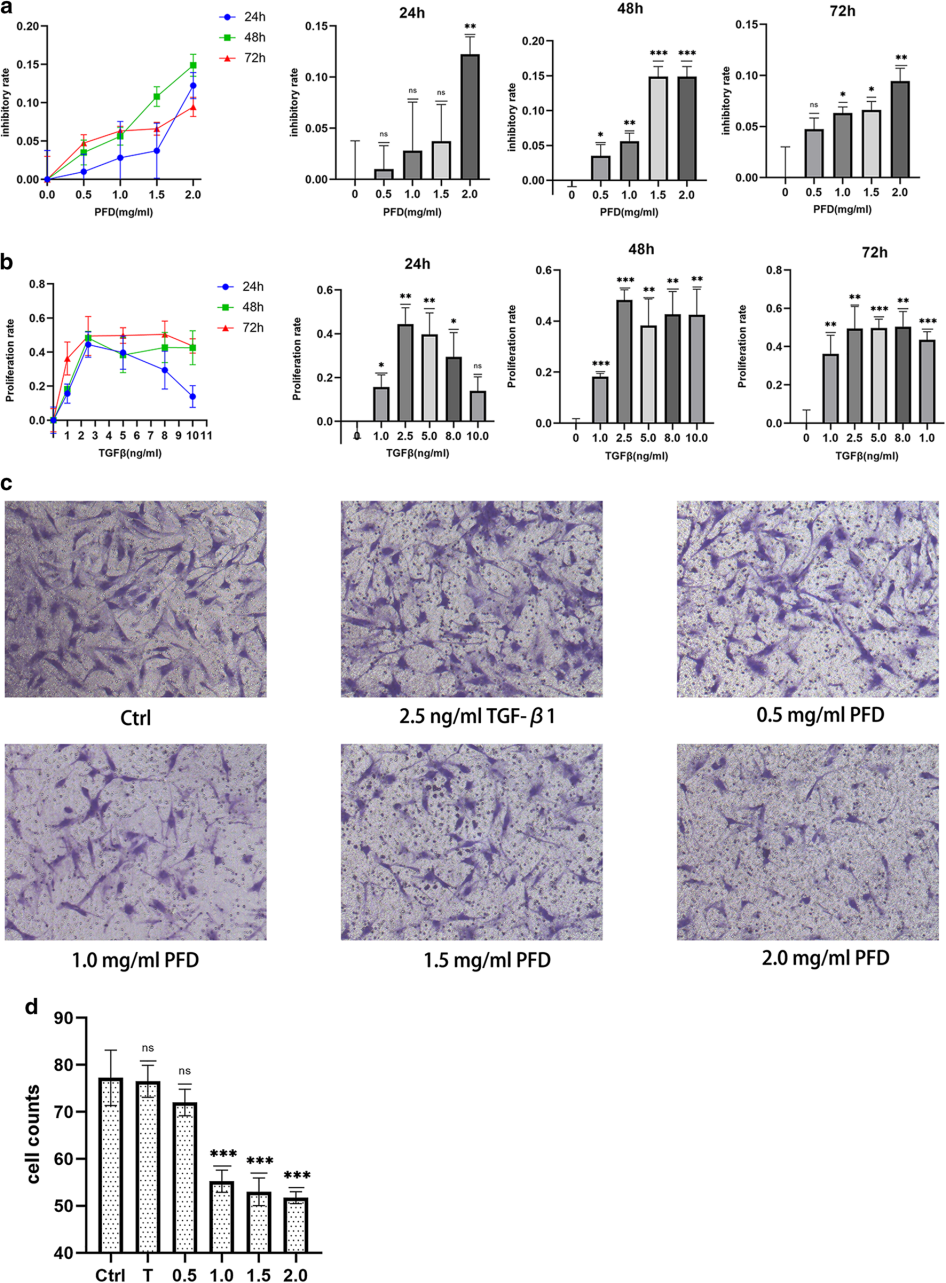
PFD inhibited the proliferation of FLSs. After 24 h, only 2.0 mg/ml PFD displayed significant inhibition compared with the 0 mg/ml (Ctrl) group. After 48 h and 72 h, inhibition by PFD was dose-dependent (**a**). TGF-β1 promoted proliferation of FLSs. TGF-β1 influenced proliferation to the greatest extent at 2.5 ng/ml compared with the 0 mg/ml (Ctrl) group at every time point (**b**). FLSs maintained a typical spindle-shaped morphology after treatment with PFD (**c**). PFD inhibited the migration of FLSs at a concentration of 1.0 mg/ml. TGF-β1 at 2.5 ng/ml did not affect the migration of FLSs (**d**). Ctrl: control group, 0 mg/ml PFD + 0 ng/ml TGF-β1; T: 2.5 ng/ml TGF-β1; 0.5: 0.5 mg/ml PFD; 1.0: 1.0 mg/ml PFD; 1.5: 1.5 mg/ml PFD;2.0: 2.0 mg/ml PFD. Data represent means ± SD. *P < 0.05, **P < 0.01, ***P < 0.001 vs. control group. (unpaired t-test)

A CCK8 cell proliferation assay was performed to determine the influence of PFD and TGF-β1 on FLSs. The results indicate that PFD inhibited the proliferation of FLSs, the degree of inhibition essentially dose-dependent at 24 h, 48 h, and 72 h (Fig. 1a). The maximum inhibition was observed at 2.0 mg/ml after 48 h. TGF-β1 facilitated FLS proliferation, the rate reaching a plateau at a concentration of 2.5 ng/ml (Fig. 1b). Therefore, 2.5 ng/ml TGF-β1 was used for subsequent FLS experiments, including analysis using Transwell assays, real-time quantitive PCR, and ELISA.

A Transwell assay was also performed to investigate the degree to which PFD and TGF-β1 affected the migration of FLSs. Crystal violet staining of the Transwell membranes indicated that treatment with PFD evidently did not affect the typical spindle-shaped morphology of the FLSs (Fig. 1c). The final results demonstrate that 2.5 ng/ml TGF-β1 (T group) did not promote the migration of FLSs (P = 0.8333), while 1.0 mg/ml PFD significantly inhibited their migration (P = 0.005, compared with the control group) (Fig. 1d).

### Effect of PFD treatment on the mRNA expression levels of COL1A1, TIMP-1, ACTA-2, IL-6, and TNF-α induced by TGF-β1

**Fig. 2:**
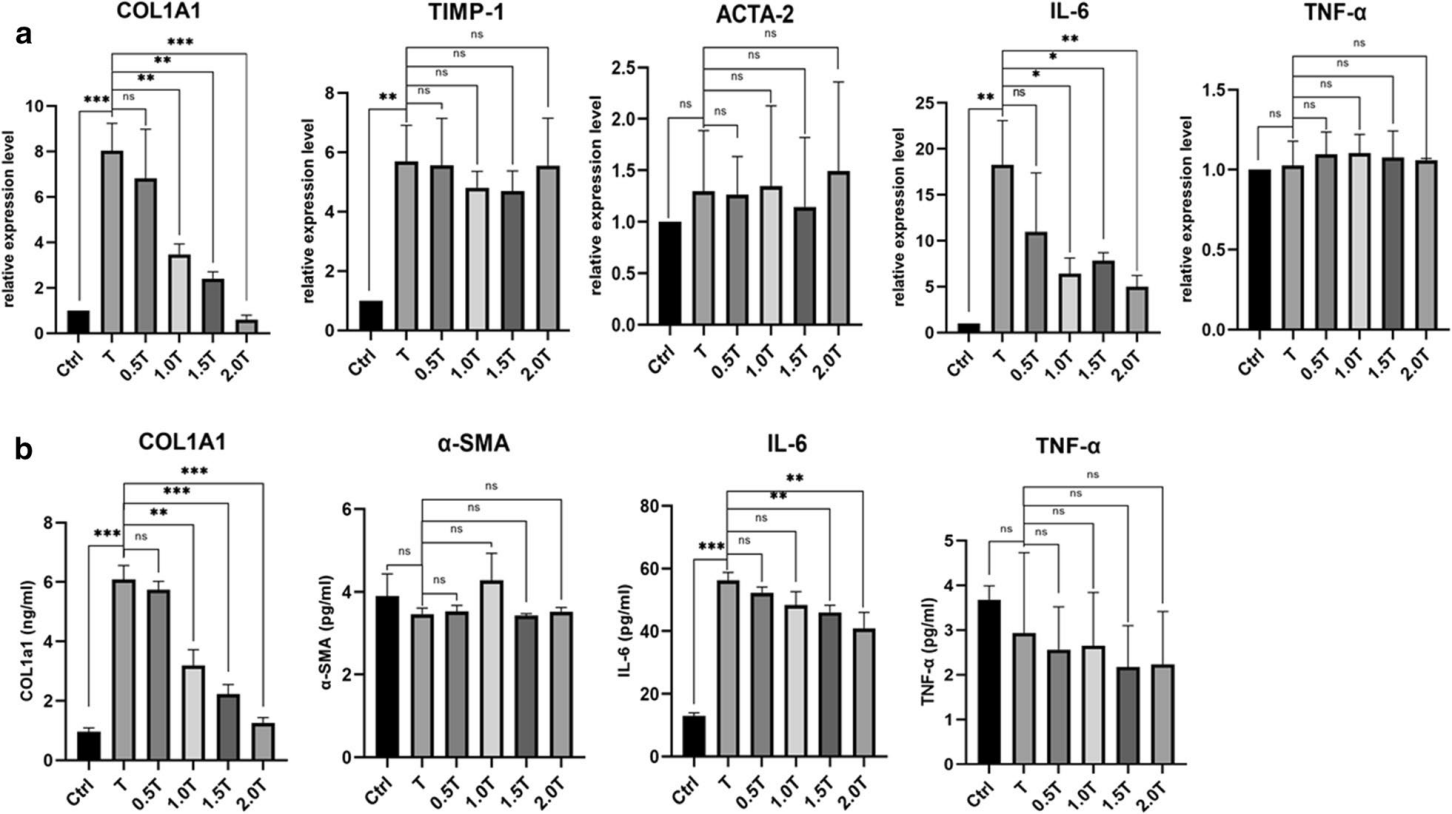
PFD reduced the expression of COL1A1 and IL-6 at both mRNA and protein levels, while PFD did not affect the expression of ACTA-2, TIMP-1, or TNF-α. Ctrl: 0 mg/ml PFD + 0 ng/ml TGF-β1; T: 0 mg/ml PFD + 2.5 ng/ml TGF-β1; 0.5 T: 0.5 mg/ml PFD + 2.5 ng/ml TGF-β1; 1.0 T: 1.0 mg/ml PFD + 2.5 ng/ml TGF-β1; 1.5 T: 1.5 mg/ml PFD + 2.5 ng/ml TGF-β1; 2.0 T: 2.0 mg/ml PFD + 2.5 ng/ml TGF-β1. Data represent means ± SD. *P < 0.05, **P < 0.01, ***P < 0.001 (unpaired t-test)

Analysis of FLSs by real-time PCR indicated that TGF-β1 (2.5 ng/ml) significantly increased mRNA expression levels of *COL1A1* (P = 0.005), *IL-6* (P = 0.0033), and *TIMP-1* (P = 0.0026) compared with the control group (no PFD or TGF-β1 in the culture medium). However, TGF-β1 did not influence the expression of *TNF-α* (P = 0.7761) or *ACTA-2* (P = 0.4329) (Fig. 2a). PFD reversed the increase in *COL1A1* and *IL-6* at 1 mg/ml and higher concentrations. In addition, PFD did not affect the expression of *TNF-α*, *TIMP-1*, or *ACTA-2*. ELISA assays further confirmed that PFD decreased the protein expression levels of IL-6 and COL1A1, while PFD or TGF-β1 had no influence on the expression of TNF-α or α-SMA (encoded by *ACTA-2*) (Fig. 2b). These results confirm that PFD inhibited the expression of COL1A1 and IL-6 that had been stimulated by TGF-β1 in vitro and established that PFD has anti-inflammatory and anti-fibrotic effects.

### Effect of PFD treatment in a model of OA

#### Real-time quantitative PCR of synovial tissue

**Fig. 3:**
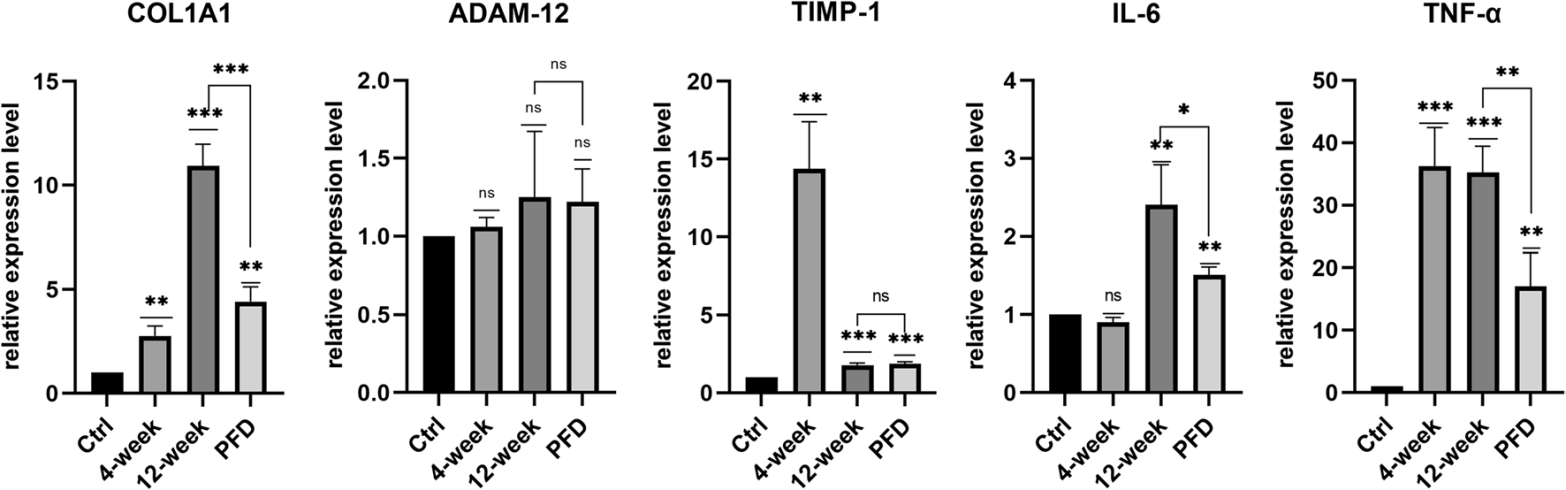
PFD decreased mRNA expression levels of *COL1A1*, *IL-6* and *TNF-α* in vivo. Data represent means ± SD. *P < 0.05, **P < 0.01, ***P < 0.001 (4-week and 12-week groups were compared with the control group; the PFD group was compared with the control and 12-week groups) (unpaired t-test)

To investigate whether treatment with PFD could ameliorate fibrosis and inflammation of the synovium and reduce or prevent the progression of osteoarthritis, a rabbit model of OA was established using a modification of the Hulth method. Gene expression levels in the synovium between the patella and femur harvested 4 and 12 weeks after surgery were measured by real-time quantitative PCR. The results indicated that after 4 weeks, the expression of fibrosis-related genes, including *COL1A1* (P = 0.0028) and *TIMP-1* (P = 0.0016) increased significantly, but not *ADAM-12*. Expression of inflammation-related genes, including *IL-6* and *TNF-α*, were also measured by real-time quantitative PCR. mRNA expression levels of *TNF-α* 4 weeks after surgery increased significantly (P = 0.0006) but the expression of *IL-6* did not change (P = 0.0550). At week 12, the expression levels of *TNF-α* (P = 0.0099) and *IL-6* (P = 0.0413) had increased significantly while 4 weeks of daily administration of 30 mg/kg PFD resulted in a significant reduction in the expression of *COL1A1* (P = 0.0009), *TNF-α* (P = 0.0099), and *IL-6* (P = 0.0413) in the PFD group of rabbits. Conversely, the expression of *ADAM-12* (P = 0.3939) and *TIMP-1* (P = 0.5114) were not modified. However, in the PFD group of rabbits, the expression of *COL1A*1 (P = 0.0011), *TIMP-1* (P = 0.003), *IL-6* (P = 0.0011), and *TNF-α* (P = 0.0068) was greater than that of rabbits in the control group (Fig. 3).

### Masson’s trichrome and immunofluorescence staining of the synovium

**Fig. 4:**
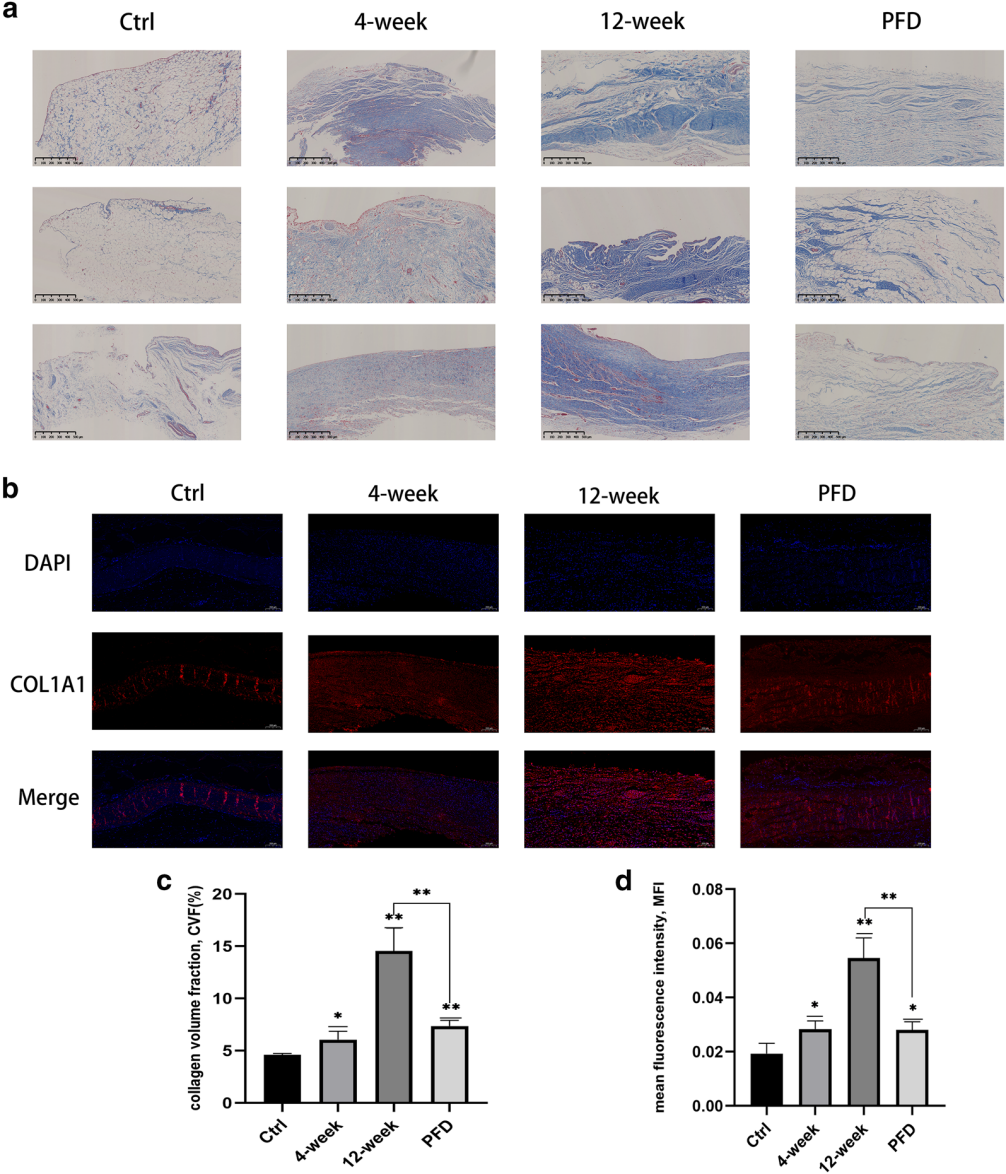
PFD attenuated the severity of synovial fibrosis (P < 0.01). Masson’s trichrome staining (**a**: scale bar-500 μm) and immunofluorescence staining (**b**: scale bar-200 μm) demonstrated that collagen deposition increased significantly in the 4-week and 12-week groups, while collagen deposition was lower in the PFD group. Quantification of collagen volume fraction (**c**) and mean fluorescence intensity (**d**) established that PFD reduced fibrosis in the synovium. Data represented means ± SD. *P < 0.05, **P < 0.01, ***P < 0.001 (the 4-week and 12-week groups were compared with the control group; the PFD group was compared with the control and 12-week groups) (unpaired t-test)

To determine the anti-fibrotic effect of PFD in vivo, the synovium of experimental rabbits was analyzed by Masson’s trichrome (Fig. 4a) and immunofluorescence staining (Fig. 4b). The depth of color in Masson’s trichrome staining indicates the quantity of collagen fibers in a section. Immunofluorescence staining indicated positive expression of type I collagen (coded by *COL1A1*). Administration of PFD resulted in decreased collagen deposition with less expression of COL1A1 than animals in the 12-week group. The volume fraction of collagen in the Masson’s trichrome stained sections (Fig. 4c) and the mean immunofluorescence intensity (Fig. 4d) were calculated using ImageJ software. The results indicate that 4 and 12 weeks after surgery, the volume of collagen was observed to have increased significantly in both Masson’s trichrome and immunofluorescence stained sections, and that PFD was effective in decreasing fibrosis in the synovium compared with animals in the 12-week group (Masson’s trichrome staining: P = 0.0057; immunofluorescence staining: P = 0.0044).

### HE staining of the synovium

**Fig. 5:**
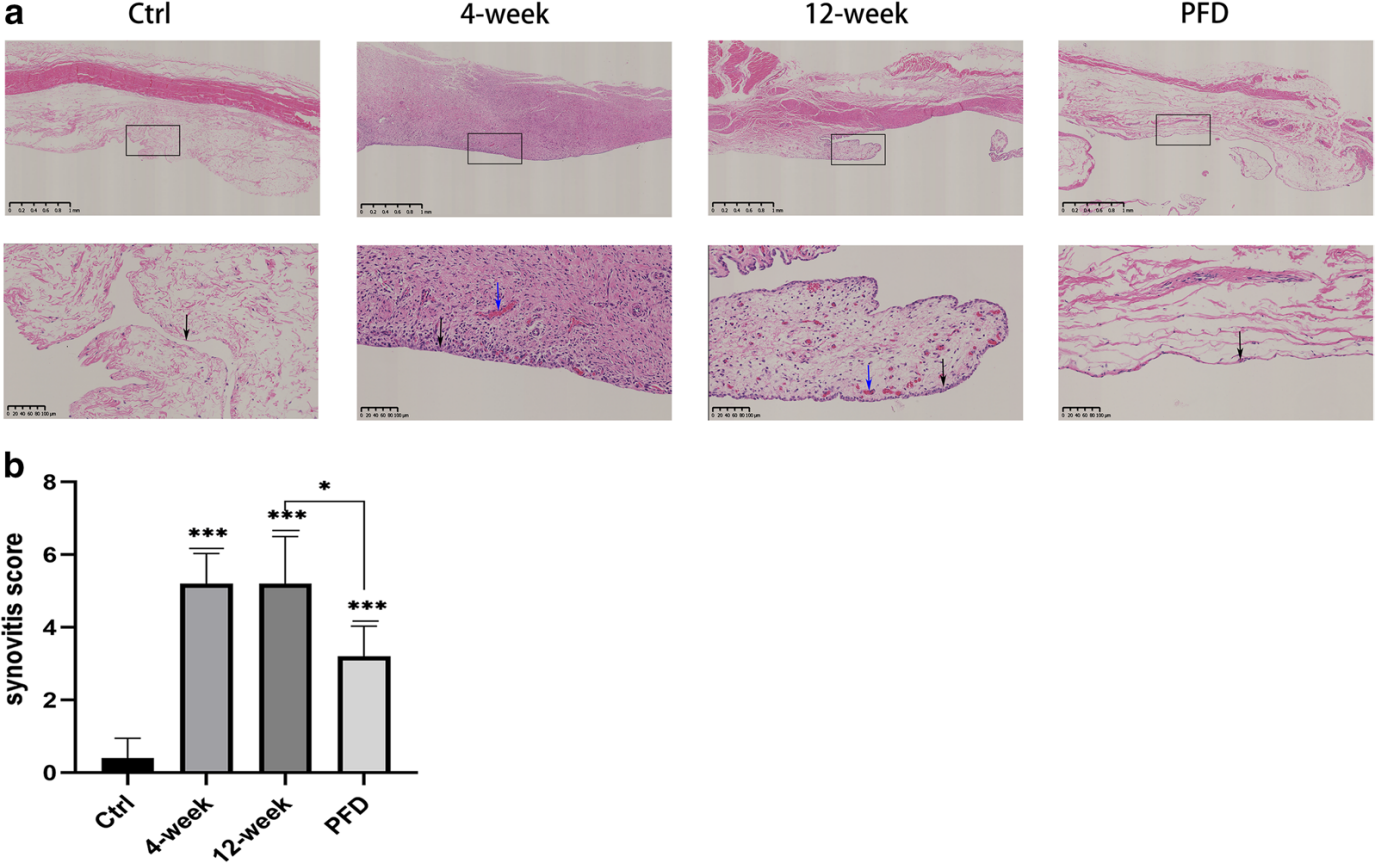
PFD reduced inflammation of the synovium. H&E staining of the synovium in different groups (**a**: scale bar of top row-1 mm; scale bar of bottom row-100 μm) and the corresponding synovitis scores (**b**). Black arrows indicate the lining of the synovium. In the control group, the lining was just 1–2 cells thick without hypertrophy or hyperplasia. Synoviocyte hypertrophy and hyperplasia were observed in the 4 and 12-week groups. Blue arrows indicate vascular proliferation, only observed in the 4 and 12-week groups. Data represent means ± SD. *P < 0.05, **P < 0.01, ***P < 0.001 (the 4 and 12-week groups were compared with the control group; the PFD group was compared with the sham and 12-week groups) (independent-samples t-test)

To determine whether PFD could reduce synovitis through its anti-inflammatory action, a synovitis score was calculated from H&E stained sections. The lining of the tissue in the control group (Fig. 5a) was 1–2 cells thick without hypertrophy or hyperplasia with very little inflammatory cell infiltration. In samples from the 4-week group, infiltration of inflammatory cells into the synovium was observed, with synoviocyte hypertrophy, hyperplasia, and vascular proliferation. In the 12-week group, the same histopathologic changes to the synovium described above were observed although increased vascular proliferation was apparent. Intervention with PFD resulted in a reduction in histopathologic changes to the synovium, such as infiltration of inflammatory cells and clearly reduced proliferation of synoviocytes.

Synovitis scores also suggested that in 4-week and 12-week animals, the synovium suffered severe synovitis, while PFD reduced inflammation compared with animals in the 12-week group (P = 0.0203) (Fig. 5b).

### Safranin O and Fast Green Staining of medial femoral condyles

**Fig. 6:**
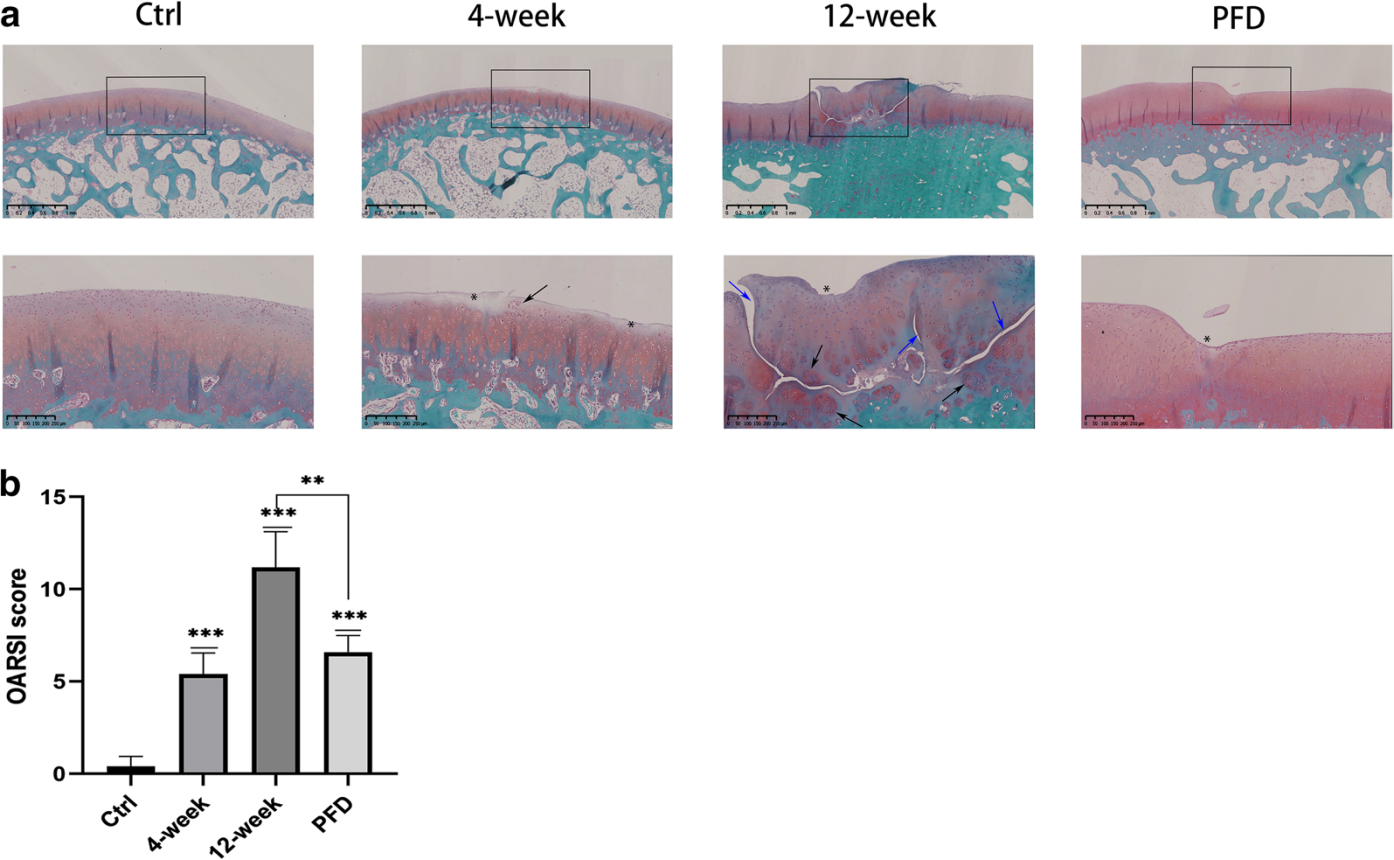
PFD delayed progression of OA. Safranin O and Fast Green staining of the medial femoral condyles from different groups (**a**: scale bar of top row-1 mm; scale bar of bottom row-250 μm) and corresponding OARSI scores (**b**). Black arrows indicate the occurrence of chondrocyte clusters and increased cell numbers, observed in samples from the 4 and 12-week groups. Blue arrows indicate fissures on the surface of the cartilage, which were significantly more severe in the 12-week group, and loss of chondrocytes. Asterisks indicate erosion of the cartilage. There was fewer fissures or chondrocyte clusters in the PFD group. OARSI scores allowed semi-quantitation of cartilage degeneration. Data represent means ± SD. * P < 0.05, ** P < 0.01, *** P < 0.001 (4 and 12-week groups were compared with the control group; the PFD group was compared with the control and 12-week groups) (unpaired t-test)

Safranin O and Fast Green Staining of the medial femoral condyles were conducted at the two time points and OARSI scores recorded, aiming to establish the effect of PFD on the progression of OA. Four weeks after surgery, Safranin O Fast Green staining indicated that the structure of the articular cartilage was slightly damaged with a loss of staining of hyaline cartilage in the superficial zone only. By week 12, severe loss of cartilage was observed. Erosion and fissures represented more than 2/3 of the entire hyaline cartilage. However, treatment with PFD altered the appearance of the structure of the cartilage, which was more complete with fewer fissures compared with the 12-week group (Fig. 6a), and OARSI scores that were significantly lower than those of the 12-week group (P = 0.0013). However, the OARSI score was nevertheless higher than that of the control group (P < 0.0001) (Fig. 6b). The results indicate that although PFD did not completely prevent the development of the disease, PFD delay the progression of OA by protecting the cartilage from erosion.

## Discussion

OA is a degenerative joint disease representing the leading cause of pain and disability worldwide [24]. However, the precise etiology and pathogenesis of OA remain unclear and no drugs are able to delay or reverse its progression. Although non-steroidal anti-inflammatory drugs (NSAIDs) are used in clinical practice, they can still cause adverse intestinal reactions and do not delay the progression of OA [25]. Therefore, identification of drugs that can prevent or delay the progression of OA remains the focus of research programs in OA.

Although degeneration of cartilage is the principal characteristic of OA, increasing numbers of studies have demonstrated that synovial pathology, including inflammation and fibrosis, plays an important role in the progression of OA [26]. Synovial inflammation provoked by pro-inflammatory cytokines, including IL-6 and TNF-α can lead to the activation and production of matrix metalloproteinases (MMPs), a disintegrins and metalloproteinases with thrombospondin motifs (ADAMTS), and inducible nitric oxide synthase (iNOS) in the joints, ultimately leading to cartilage destruction [27, 28]. Synovial fibrosis in OA is associated with joint pain and stiffness, with the expression of TGF-β considered the most critical cytokine. TGF-β signaling is pivotal in fibrogenesis and thus TGF-β1 is widely used to model fibrosis in vitro [29–32]. In the present study, we explored whether TGF-β1 influences the proliferation of FLS and found that a concentration of 2.5 ng/ml TGF-β1 promoted the greatest FLS proliferation. This concentration has also been used in other studies of TGF-β1-induced fibrosis of fibroblasts [31].

The present research aimed to identify a synovium-targeting therapy for OA. As synovial fibrosis and inflammation is critical in the progression of OA, the anti-inflammatory and anti-fibrotic properties of PFD were investigated both in vitro and in vivo. The precise mechanisms of action of PFD remain undefined, although it is approved by the FDA for the treatment of idiopathic pulmonary fibrosis [33]. Previous studies have confirmed that PFD decreases autocrine expression of TGF-β and MMP-1 in human pterygium fibroblasts [34] and inhibits the production of collagen I in human intestinal fibroblasts[31]. PFD decreases the deposition of hydroxyapatite by osteoblasts in a dose-dependent manner, which may inhibit osteophyte formation [35]. Furthermore, PFD can reduce subchondral bone loss and fibrosis following murine knee cartilage injury [36]. However, the influence of PFD on FLSs from OA and its efficacy in the treatment of OA remains incompletely characterized.

FLSs were harvested from patients undergoing total knee replacement surgery. We found that TGF-β1 was effective in increasing the expression of fibrosis-related genes, including *COL1A1* and *TIMP-1*. PFD decreased the expression of *COL1A1* significantly (P < 0.05) in a dose-dependent manner. Protein expression levels of COL1A1 were verified by ELISA. The results indicate that PFD at a concentration of 1.0 mg/ml reduced the expression of COL1A1 induced by TGF-β1. The expression of IL-6 was also greater after stimulation by TGF-β1. PFD reversed this effect at a concentration of 1.5 mg/ml. However, neither TGF-β1 nor PFD influenced the expression of TNF-α. PFD displayed anti-fibrotic and anti-inflammatory properties as a result of down-regulation of COL1A1 and IL-6, respectively, in FLSs.

In the present study, a modified Hulth method was selected to establish an OA model of the knee. According to previous studies, such a method causes severe inflammation [28] and after 4 weeks, knee joints have been shown to undergo early pathological changes characteristic of OA [37]. The present study also found that, by 4 weeks post-surgery, the cartilage had begun to manifest slight erosion. Therefore, this time point was used for intervention in the PFD group using PFD. We anticipated that PFD would display anti-fibrotic and anti-inflammatory properties, thus preventing the progression of OA.

The synovium between the patella and femur was examined using immunofluorescence and Masson’s trichrome staining, and qRT-PCR to evaluate the severity of synovial fibrosis after surgery. Expression of fibrosis-related genes, including *COL1A1*, *ADAM-12*, and *TIMP-1* increased significantly. The collagen volume fraction in Masson’s trichrome stained sections and the mean fluorescence intensity by immunofluorescence indicated that collagen deposition increased after 4 and 12 weeks compared with the control group. The results confirmed that fibrosis becomes increasingly serious over time. Intervention with PFD resulted in a decrease in COL1A1 expression and collagen deposition.

The anti-inflammatory properties of PFD were investigated by calculating the synovitis score of H&E sections. H&E staining of the synovium in the 4-week and 12-week group indicated clearly signs of synoviocyte hypertrophy, hyperplasia, matrix edema and inflammatory cell infiltration compared with the control group. Compared with the 12-week group, the PFD group had fewer inflammatory cells and the severity of hypertrophy, hyperplasia, and edema was also significantly lower. Because PFD was hypothesized to decrease the severity of fibrosis and inflammation in the synovium, Safranin O and Fast Green staining was conducted, from which an OARSI score was calculated to determine whether PFD was able to delay the progression of OA. Compared with the 12-week group, the structure of the cartilage from the PFD group was more complete with a lower OARSI score which suggested delayed progression of OA.

The present study has a number of potential limitations. Firstly, the pathophysiology and pathogenesis of OA induced by the modified Hulth method are complex and unclear. It was confirmed in the present study that synovitis was clearly elevated in the OA synovium in groups in which the modified Hulth method was used, at all time points. Although previous studies have shown that cytokines produced by an inflammatory synovium, including IL and TNF, are able to induce a degradative cascade leading to joint damage [38], it was not possible to determine which initiated the disease and whether synovitis was decisive in promoting the progression of OA. Although the study established that PFD decreased the expression of proinflammatory cytokines and reduced fibrosis in the synovium, the specific mechanism by which PFD postponed the progression of OA remains unclear. Furthermore, the mechanism by which PFD influences the function of chondrocyte remains unclear. Although the study established that PFD reduces subchondral bone loss and fibrosis in murine knee cartilage [36], whether PFD is able to promote cartilage repair and its production requires additional research. Finally, OA induced by the modified Hulth method is posttraumatic and different from the degenerative OA occurring in the elderly. Therefore, the efficacy of PFD requires investigation in different models of OA for verification.

## Conclusion

Taken together, the study demonstrated that PFD displays anti-inflammatory and anti-fibrotic effects in the OA synovium. Meanwhile, PFD delayed the progression of OA in the rabbit OA model. The research provides evidence for the potential of synovium-targeted therapy in OA.

## Data Availability

The datasets used and analyzed during the current study are available from the corresponding author on reasonable request.

## Abbreviations

PFD: Pirfenidone
OA: Osteoarthritis
FLSs: Fibroblast-like synoviocytes
TKA: Total knee arthroplasty
CCK-8: Cell counting Kit-8 assay
ELISA: Enzyme-linked immunosorbent assay
OARSI: Osteoarthritis Research Society International’s histopathology grading system
