# Correction to: Pirfenidone attenuates synovial fibrosis and postpones the progression of osteoarthritis by anti-fibrotic and anti-inflammatory properties in vivo and in vitro

**DOI:** 10.1186/s12967-021-03106-8

**Published:** 2021-10-18

**Authors:** Qilu Wei, Ning Kong, Xiaohui Liu, Run Tian, Ming Jiao, Yiyang Li, Huanshuai Guan, Kunzheng Wang, Pei Yang

**Affiliations:** Bone and Joint Surgery Center, The Second Affiliated Hospital of Xi’an Jiaotong University, Xi’an 710004, China

## Correction to: BMC Ecol Evol (2021) 19:157 https://doi.org/10.1186/s12967-021-02823-4

The authors of the original article [1] have found out after publication that there was an error in Fig. 4a. The left image in the third row is replaced accordingly. The correct (Fig. 1) and incorrect figure (Fig. 2) are shown in this correction article.

**Fig. 1:**
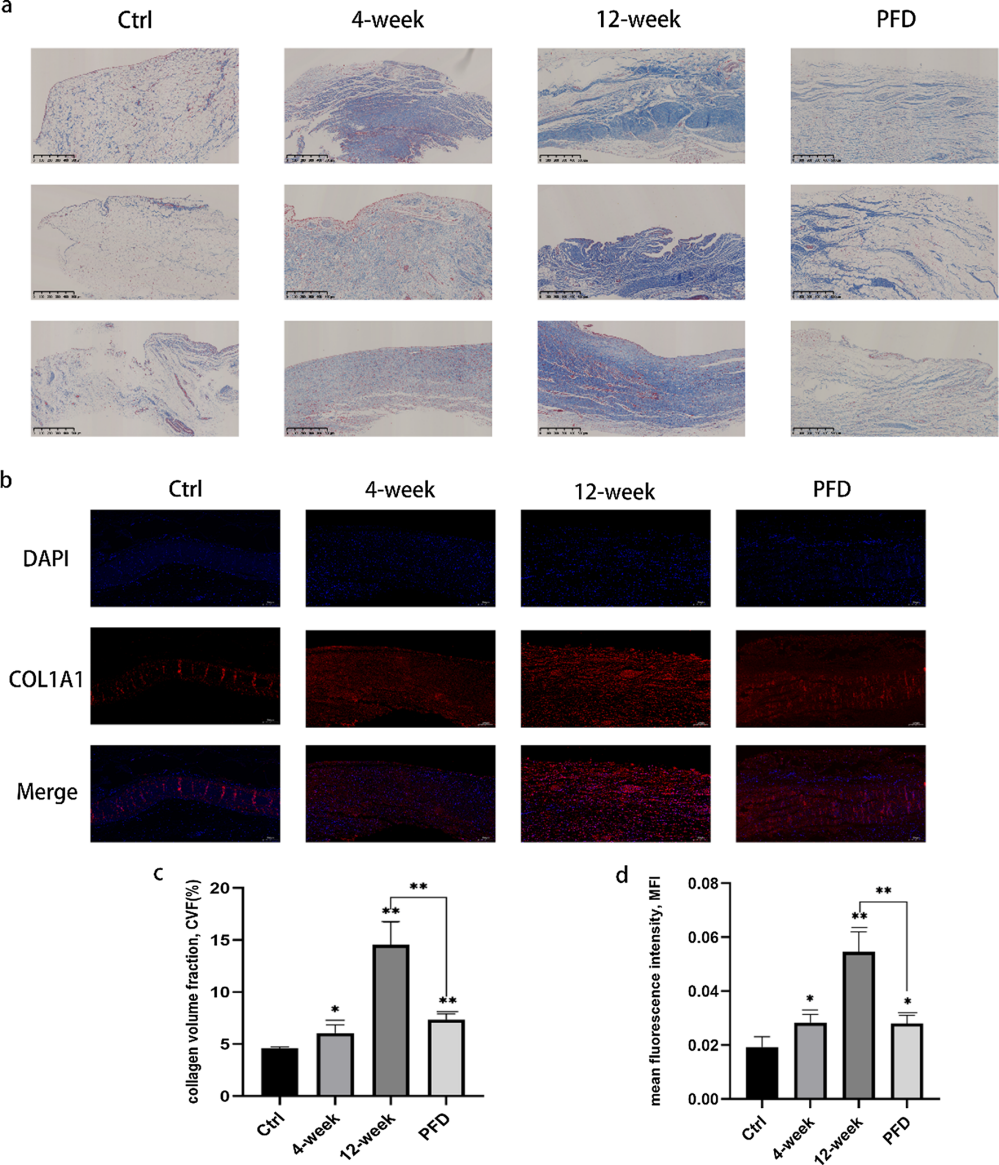
Correct version of Fig. 4

**Fig. 2:**
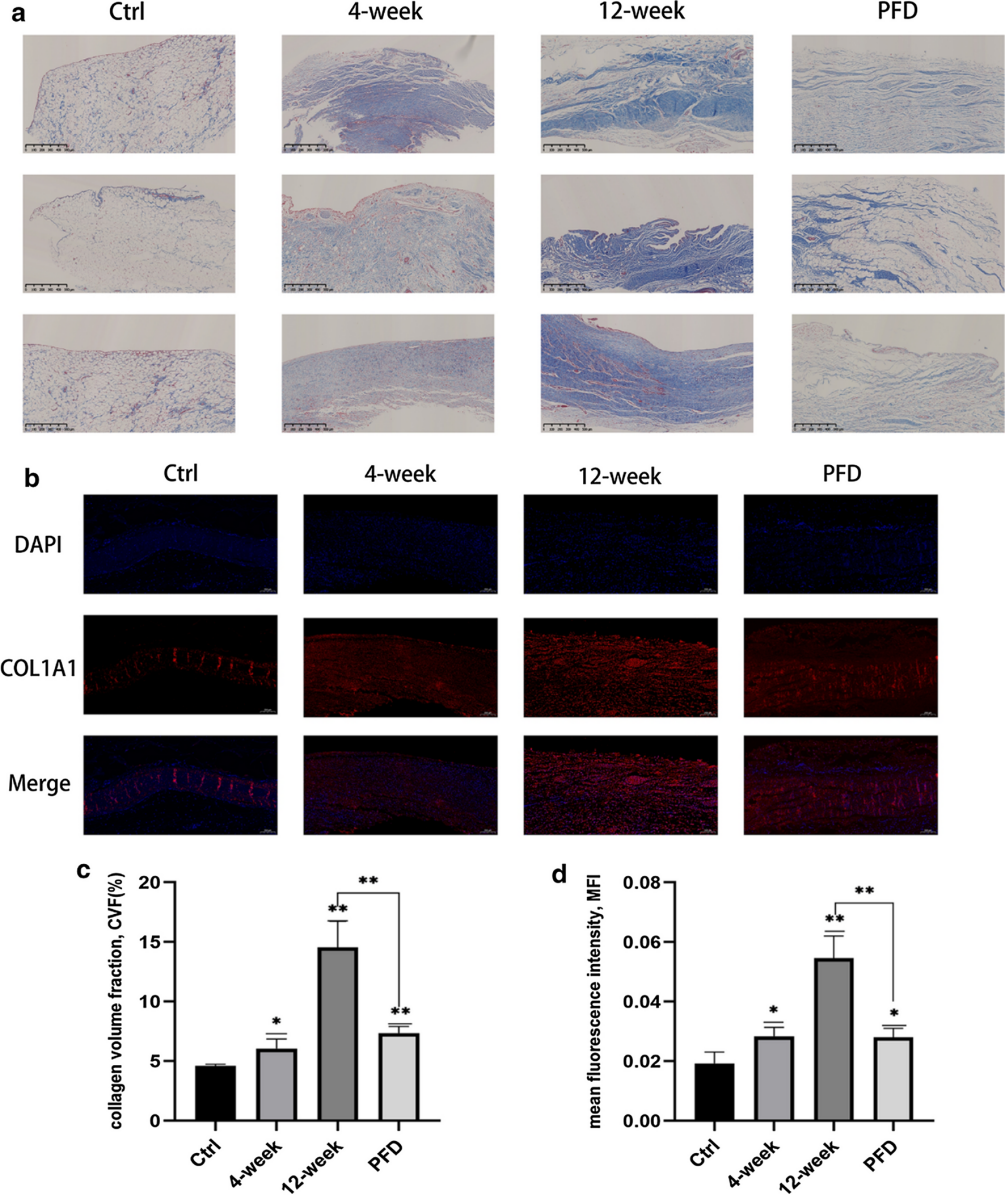
Incorrect version of Fig. 4 as originally published

This correction does not affect either results or conclusions. We apologize for the inconvenience to the readers.

## References

[1] Wei Q, Kong N, Liu X, Tian R, Jiao M, Li Y, Guan H, Wang K, Yang P (2021). Pirfenidone attenuates synovial fibrosis and postpones the progression of osteoarthritis by anti-fibrotic and anti-inflammatory properties in vivo and in vitro. J Transl Med.

